# Dataset on psychosocial risk factors in cases of fatal and near-fatal physical child abuse

**DOI:** 10.1016/j.dib.2017.07.003

**Published:** 2017-07-11

**Authors:** Mary Clyde Pierce, Kim Kaczor, Deborah Acker, Tina Webb, Allen Brenzel, Douglas J. Lorenz, Audrey Young, Richard Thompson

**Affiliations:** a Department of Pediatrics, University of Louisville, 571 S. Floyd St., Louisville, KY 40202, USA; b Department of Community Based Services, Cabinet for Health and Family Services, Commonwealth of Kentucky, 275 East Main St, Frankfort, KY 40621, USA; c Division of Protection and Permanency, Cabinet for Health and Family Services, Commonwealth of Kentucky, 275 East Main St., Frankfort, KY 40621, USA; d Department of Psychiatry, University of Kentucky College of Medicine, 245 Fountain Court, Lexington, KY 40509, USA; e Department of Bioinformatics and Biostatistics, School of Public Health and Information Sciences, University of Louisville, 485 E. Gray St., Louisville, KY 40202, USA; f Northwestern University Feinberg School of Medicine, 225 E. Chicago Ave., Chicago, IL 60611, USA; g Richard H. Calica Center for Innovation in Children and Family Services, Juvenile Protective Association, 1707 N Halsted St, Chicago, IL 60614 USA

## Abstract

This article presents the psychosocial risk factors identified in the cases of 20 children less than four years of age who were victims of fatal or near-fatal physical abuse during a 12 month period in the Commonwealth of Kentucky. These data are related to the article “History, injury, and psychosocial risk factor commonalities among cases of fatal and near-fatal physical child abuse” (Pierce et al., 2017) [1].

**Specifications Table**

**Table:** 

Subject area	*Medicine, Psychology*
More specific subject area	*Child Abuse*
Type of data	*Table*
How data was acquired	*Retrospective case review*
Data format	*Analyzed*
Experimental factors	*State records including medical, social, and legal documents were obtained from the Kentucky Cabinet for Health and Family Services.*
Experimental features	*Abstracted data regarding psychosocial risk factors pertaining to caregivers in the child׳s environment*
Data source location	*Commonwealth of Kentucky, USA*
Data accessibility	*Data are available within this article.*

**Value of the data**

• These data reveal commonalities among cases of fatal and near-fatal child abuse and highlight the high prevalence of psychosocial risk factors among caregivers.
• By identifying indicators of maltreatment that were present *prior to* each child׳s fatal or near-fatal event, these data may assist medical providers, psychologists, social workers, and legal representatives in collaboratively assessing risk for maltreatment and intervening to prevent future injury.
• These data open doors for further exploration of risk factors for maltreatment, and may guide future research efforts to better inform strategies for child abuse prevention.

## 1 Data

Psychosocial risk factors identified in 20 cases (10 fatal, 10 near-fatal) of physical child abuse are presented in Table 1. An X indicates the presence of a given risk factor among one or more caregivers in the child׳s environment.

**Table 1:** Psychosocial risk-factors identified.

	**Fatalities**										**Near-fatalities**									
**Risk-factor**	**1**	**2**	**3**	**4**	**5**	**6**	**7**	**8**	**9**	**10**	**11**	**12**	**13**	**14**	**15**	**16**	**17**	**18**	**19**	**20**
**Protective services history**																				
Perpetrator prior victim	X							X		X					X	X				X
Perpetrator/parent prior reports		X	X		X	X	X	X	X	X		X	X	X		X	X	X		
**Social factors**																				
Recent/frequent moves		X			X			X	X											
Recent change to household composition or childcare	X	X			X				X		X			X						
Reported financial difficulties	X	X				X	X					X								X
Suicide attempts parent/perpetrator	X																			
Substance use history	X	X	X				X	X									X	X	X	
Diagnosed/identified mental illness	X	X			X	X		X	X					X						X
Caregiver developmental delay/disability			X					X							X			X		
Visitation or custody dispute			X	X		X			X	X										
Complaints of baby behavior/colicky/crying					X	X		X		X	X		X					X		X
Developmentally inappropriate expectations/negative attributes/not protective		X	X		X			X		X	X	X	X	X	X	X	X	X	X	X
**Violence**																				
Threats/violence among family members	X	X	X	X			X		X			X		X		X				
Domestic violence	X	X	X	X	X		X	X	X	X		X		X	X	X	X	X	X	
Orders of protection		X	X		X			X				X		X						
Mandated anger management classes			X						X											
**Criminal history**																				
Perpetrator Criminal history	X	X	X	X	X	X	X	X	X		X	X		X		X	X	X	X	
Non-offending parent/partner criminal history	X		X		X	X	X			X			X	X	X	X	X			
Incarceration – parent/partner or family member		X	X							X					X		X			X

## 2 Experimental design, materials and methods

The Institutional Review Boards at the University of Louisville and the Kentucky Cabinet for Health and Family Services (KY CHFS) approved this research. Our retrospective record review included 20 children younger than four years of age who had been victims of fatal (*n*=10) or near-fatal (*n*=10) child abuse in the Commonwealth of Kentucky [1]. We utilized the Kentucky Revised Statues [KRS 600.020 (37)] definition of near-fatality: an injury that places a child in serious or critical condition as certified by a physician. The following documents were reviewed when available: medical records associated with all medical visits prior to and including the fatal/near-fatal event, social service evaluations prior to and including the fatal/near-fatal event, legal proceedings, criminal histories of caregivers, and autopsy findings (when applicable). Three independent investigators reviewed and abstracted data from each de-identified case simultaneously, noting the presence or absence of previously identified psychosocial risk factors in each case.
