# Supplementary material for: Dataset on psychosocial risk factors in cases of fatal and near-fatal physical child abuse
Source: Data Brief. 2017 Jul 11;14:107–9. doi: 10.1016/j.dib.2017.07.003 (PMC5537379; doi:10.1016/j.dib.2017.07.003)
Supplement: Supplementary file 1 — Supplementary material [file mmc1.docx]

Regarding manuscript: “Dataset on Psychosocial Risk Factors in Cases of Fatal and Near-Fatal Physical Child Abuse”

None of the authors have any conflict of interest as per the conflict of interest guideline provided by Elsevier website.
